# Review for the generalist: evaluation of pediatric foot and ankle pain

**DOI:** 10.1186/1546-0096-6-6

**Published:** 2008-04-09

**Authors:** Kristin M Houghton

**Affiliations:** 1 Division of Rheumatology, British Columbia Children's Hospital, Vancouver, Canada; 2 Department of Pediatrics, University of British Columbia, Vancouver, Canada

## Abstract

Foot and ankle pain is common in children and adolescents. Problems are usually related to skeletal maturity and are fairly specific to the age of the child. Evaluation and management is challenging and requires a thorough history and physical exam, and understanding of the pediatric skeleton. This article will review common causes of foot and ankle pain in the pediatric population.

## Background

Foot and ankle problems are common in the pediatric population. Problems can be related to skeletal maturity and are fairly specific to the age of the child. An awareness of congenital anomalies, developmental variation, skeletal maturation and lower extremity alignment will aid the physician in evaluation and management. This article will review common causes of foot and ankle pain in the pediatric population. It is not meant to be an exhaustive review and will not review acute traumatic fractures.

### Clinical history

Most foot and ankle pain in the active pediatric population are associated with minor trauma or repetitive stress combined with abnormal biomechanics of the foot and lower extremity. Older children may isolate pain to a specific site whereas toddlers are more likely limp or refuse to bear weight. Often there is no clear history of traumatic event. There are numerous, non-traumatic diseases that masquerade as injuries.

The clinical history should include a thorough description of the pain characteristics (location, character, onset, duration, change with activity or rest, aggravating and alleviating factors, night pain); trauma (acute macrotrauma, repetitive microtrauma, recent/remote); mechanical symptoms (locking, catching, clicking, instability, worse during or after activity); inflammatory symptoms (morning stiffness, swelling); neurological symptoms (weakness, altered sensation); gait (limp, altered weight bearing); effects of previous treatments and the current level of function of the child. Location of pain is the most important historical factor in aiding diagnosis.

A history of previous injury and/or surgery, neurological disorder, chronic inflammatory joint disease or bleeding diathesis is significant. Family history of orthopedic, neurologic or rheumatic disease is also important.

### Anatomy and physical examination

**Figure 1:**
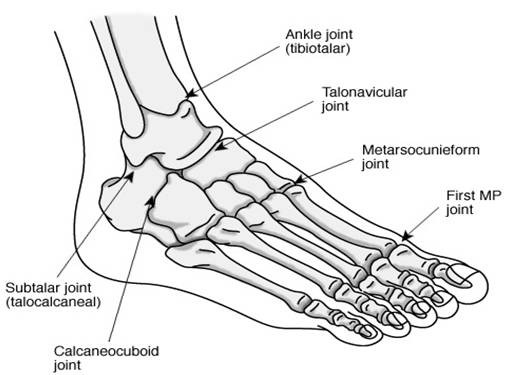
**Anatomy of the foot and ankle**. (Picture courtesy of Allan McGavin Sports Medicine Centre patient handouts).

The basic anatomy of the foot and ankle is shown in Figure 1. Foot and ankle pain can be localized to the forefoot, midfoot, hindfoot or ankle. The forefoot includes the metatarsals, phalanges and sesamoids; the midfoot includes the tarsal bones, (navicular, cuboid and three cuneiforms); and the hindfoot includes the talus and calcaneus. The Lisfranc joint includes the five metatarsophalangeal (MTP) joints and separates the midfoot and forefoot and the talonavicular and calcaneocuboid articulation (Chopart joint) separates the midfoot from the hindfoot. The subtalar joint separates the talus and calcaneus. Movement at the Chopart joint includes supination and pronation. Movement occurs in triplanar patterns at the subtalar joint: supination (inversion, adduction, plantarflexion) and pronation (eversion, abduction, dorsiflexion) The main functions of the foot are to support the body, absorb shock from ground reaction forces, and to provide a rigid lever for gait.

The ankle is a simple hinge joint composed of the tibia and fibula, which both articulate with the talus. Movement occurs in triplanar patterns: dorsiflexion and plantarflexion, dorsiflexion with eversion and abduction, and plantarflexion with inversion and adduction. Static stability is provided by the lateral ligament complex (anterior talofibular, calcaneofibular and posterior talofibular ligaments) and the medial deltoid ligament (originates from the distal tibia). Dynamic stability is provided by the peroneus brevis and longus muscles laterally (everts foot) and the posterior tibialis muscle medially (inverts foot). The main functions of the ankle are to provide stability for weight bearing and to allow mobility of the foot [1].

During the clinical assessment the physician should try and reproduce the patient's foot or ankle pain through palpation and manipulation. Biomechanical examination is important in determining any potential predisposing or contributing factors. This should include an assessment for genetic predisposing factors such as excessive stiffness, loose-jointedness, pes planus, pes cavus, and/or increased or decreased muscle tone. Functional biomechanics should be assessed by evaluation of gait, and maneuvers such as jumping, hopping (single and double-leg), cutting and figure of 8's running. Most causes of foot and ankle pain are unilateral, allowing comparison to the unaffected side. The lumbar spine, ipsilateral hip and knee should always be examined.

#### 1. Observation

Standing [Pelvis heights, lower limb alignment, hindfoot position (varus, valgus), subtalar position (inversion, eversion), forefoot position (adduction, abduction), foot arch (cavus, planus), toes (claw toes, overriding fifth toe, Morton's foot with first ray shorter than the second), swelling, redness.]

Supine or sitting [Lower limb lengths and alignment, hindfoot position, forefoot position, foot arch, alteration of callus formation due to altered weight bearing, corns, plantar warts, onchocryptosis.]

#### 2. Palpation

Surface anatomy is best appreciated with the patient sitting. The foot and lower leg are stabilized by holding the foot around the calcaneus. It is important to palpate specific structures. The point of maximal tenderness should be correlated with the underlying bone or soft tissue anatomy. Palpate both malleoli and their respective physes, which lie 1 inch (2.5 centimeters) from the tip of each malleoli. Palpate over the joints, tarsal bones, metatarsals, phalanges and along the course and attachments of ligaments and tendons. Ankle joint effusion and tenosynovitis may be palpable.

#### 3. Range of motion

The resting position of the foot and ankle is slight ankle plantarflexion (10°), subtalar neutral.

Passive movements [Ankle dorsiflexion (10–20°; if patient is unable to dorsiflex ankle with knee extended but can dorsiflex with knee flexed the gastrocnemius is the cause of limited range whereas if range limitation is the same with knee extended and flexed soleus is involved), ankle plantarflexion (50°), subtalar eversion (15–20°), subtalar inversion (35–40°), forefoot adduction (20°), forefoot abduction (10°), first MTP flexion (45°), first MTP extension (70–90°), motion of lesser toes.] Range of motion decreases with age, especially at the subtalar joint with eversion (10°) and inversion (20°) in adulthood.

#### 4. Special tests

1) Tests for rigid or flexible flat feet [Observe patient's medial longitudinal arch standing, standing on his/her toes, and sitting. Presence of an arch while on one's toes or sitting and absent with standing suggests flexible flat feet. Absence of an arch in all positions suggests rigid flat feet.]

2) Thigh foot angle [Measure of tibial torsion. Patient prone with knee flexed to 90° and foot in relaxed position; measure angle between the axis of the thigh and foot. The thigh foot angle rotates laterally with increasing age; the upper limit of normal is 30°. Negative values are common in infants. Negative and positive values beyond the normal range are referred to as medial (internal) tibial torsion and lateral (external) tibial torsion respectively.]

3) Forefoot adduction correction test [Patient supine or prone. The lateral border of the foot is usually straight. If forefoot adduction is correctable to neutral or beyond with abduction it is "correctable" and if "fixed" it likely requires orthopedic intervention and casting.]

4) Anterior drawer and talar tilt tests for lateral ankle sprains. [The anterior drawer tests the stability of the anterior talofibular ligament (ATFL) and the talar tilt the stability of the calcaneofibular (CFL) ligament. It is normal to have a small amount of movement but pain, a soft endpoint and marked difference from side to side all indicate injury to the ligament. For the anterior drawer the ankle is relaxed in a neutral or slightly plantar flexed position, the tibia and fibula are stabilized with one hand while the other hand attempts to pull the talus forward out of the ankle mortise. The talar tilt is performed by inverting the calcaneus with the ankle in neutral position.]

5. External rotation test for "high" ankle sprains (injuries to the anterior tibiofibular ligament) [Stabilize the lower leg in neutral with one hand and abduct the foot with the other hand. The test is positive if it produces pain.]

#### 5. Flexibility

The ankle, subtalar, forefoot and MTP joints should be moved thru active range and then placed thru full passive range of motion. Muscles that span two joints are important for functional range of motion and should be tested independently (gastrocnemius muscles).

#### 6. Strength

Ankle dorsiflexion [tibialis anterior, extensor hallucis longus, extensor digitorum longus]

Ankle plantarflexion [gastrocnemius, soleus, flexor hallucis longus, flexor digitorum longus]

Subtalar inversion [tibialis posterior]

Subtalar eversion [peroneus longus and brevis]

First MTP dorsiflexion [extensor hallucis longus]

First MTP plantar flexion [flexor hallucis longus]

#### 7. Spine, hip and knee joint exam

Range of motion. Further detailed exam as appropriate.

#### 8. Neurovascular exam

Achilles reflex [S1].

Sensation of the lower leg in major dermatomes.

Posterior tibialis and dorsalis pedis artery pulsations.

#### 9. Gait

Normal heel toe gait requires ankle dorsiflexion of at least 10°, subtalar pronation and supination of 5° and 1st MTP dorsiflexion of 40°. In normal walking, 60% of time is spent in the stance phase (25% double stance) and 40% in the swing phase [2]. The stance phase includes heel strike, foot flat, heel rise and toe off. Altered gait may be due to truncal or lower limb malalignment, lower extremity muscle weakness, joint instability, limited joint range of motion, and/or pain.

Toe walking [plantarflexion and lesser toe motion]

Heel walking [dorsiflexion]

Lateral borders of feet [inversion]

Medial borders of feet [eversion]

### Investigations

Laboratory tests are almost never necessary in evaluating a patient with foot or ankle pain. CBC, ESR or CRP, blood and joint cultures should be done if infection is suspected. Arthritis is a clinical diagnosis; anti-nuclear antibody (ANA), rheumatoid factor and HLA-B27 are helpful in classification and treatment but not diagnosis. CBC, peripheral smear should be done if hematological malignancy is suspected.

### Imaging

#### Radiographs

Standard ankle views include the anteroposterior (AP), lateral and mortise projections. The mortise view is taken anterior to posterior with the leg internally rotated 10–20 degrees to allow visualization of the talus and joint space of the ankle [3].

The Ottawa ankle rules (Table 1) were developed for patients over age 18 but studies suggest they can be used for children age 10 and older [4]. This may decrease the need for ankle and foot x-rays [5,6]. Weight bearing AP, lateral, oblique and axial view (talocalcaneal coalition) of the feet is useful for investigation of painful rigid flatfoot (tarsal coalition). Calcaneonavicular coalition is best seen on the 45-degree oblique radiograph.

**Table 1:** The Ottawa ankle rules (4).

*An ankle x-ray series is only necessary if there is pain in the malleolar zone and any of the following:*
1. Bone tenderness at the posterior edge or tip of the lateral mallelous, or
2. Bone tenderness at the posterior edge or tip of the medial mallelous, or
3. Inability to weight bear both immediately and in the emergency department

*A foot x-ray series is only necessary if there is pain in the midfoot zone and any of the following:*

1. Bone tenderness at the base of the 5^th ^metatarsal, or
2. Bone tenderness at the navicular bone, or
3. Inability to weight bear both immediately and in the emergency department

#### Computed tomography (CT)

Many bones may not be completely ossified so CT is often needed to provide additional bony and cartilage detail. CT is useful in identifying bony tarsal coalition not visualized on radiographs. CT findings may show subtle trabecular irregularity associated with bone necrosis when plain radiographic findings are normal.

#### Magnetic resonance imaging (MRI)

MRI provides increased soft tissue contrast allowing evaluation of muscles, tendons, ligaments, retinaculum, neurovascular bundle, bursa and synovium. MRI also allows more detailed evaluation of articular and physeal cartilage, subchondral bone, periosteum, and bone marrow elements [7]. Intravenous injection of gadolinium is not routine but is useful to delineate synovial enhancement [8].

MRI is useful in the diagnosis of foot and ankle pain but anatomic localization on clinical exam is necessary to increase the specificity of imaging. MRI is very sensitive; high-signal T2-weighted bone marrow changes can be found in both bone marrow edema and normal hematopoietic marrow [9].

#### Technetium bone scan

Bone scan identifies areas of increased osteoblastic activity and can help localize subtle areas of bone injury such as early stress fracture. However, because of the many bones in the foot, even with SPECT (single-photon emission computed tomography) it is often difficult to localize the signal to a particular bone.

### Acute injuries

In contrast to adults, children have relatively stronger ligaments than bone or cartilage. Trauma results in growth plate fractures (Salter-Harris) more commonly than sprains. It is important to "rule out a fracture" before thinking of a "sprain" in skeletally immature athletes. Children's forefeet are flexible and fairly resilient to injury. Metatarsal and phalangeal fractures are often difficult to recognize because of multiple growth centers [10]. A thorough examination and ancillary diagnostic imaging is often necessary for accurate diagnosis and appropriate management. Acute traumatic fractures will not be covered in this review.

#### Lateral ligament complex sprain

**Figure 2:**
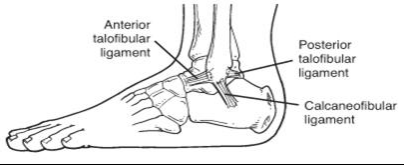
**Anatomy of the lateral ankle ligament complex**.(Picture courtesy of Allan McGavin Sports Medicine Centre patient handouts).

Lateral malleloar pain is common following an ankle injury. The mechanism of injury of most ankle sprains is inversion. The anterior talofibular ligament (ATFL) is the most commonly injured ligament. The calcaneofibular (CFL) and posterior talofibular (PTFL) are involved in more serious injury. (Figure 2) Children usually complain of pain over the lateral mallelous and difficulty weight bearing. On examination, there may be anterolateral swelling and/or bruising, tenderness over the lateral ligaments, instability (positive drawer test) and difficulty bearing weight. Bone tenderness and inability to weight bear suggests possible fracture. The Ottawa ankle rules (Table 1) were developed as a clinical practice guideline for obtaining foot and ankle radiographs in persons over the age of 18 years and have excellent sensitivity for detecting ankle and midfoot fractures [4,11]. The Ottawa ankle rules don't account for growth plates and can be applied to younger individuals who are skeletally mature [5]. Younger children are more likely to have growth plate fractures (Salter-Harris) than sprains, requiring a high index of suspicion of more complicated diagnoses following inversion injury. Other injuries to consider include interruption of the syndesmotic ligament between the tibia and fibula ('high ankle sprain') and fracture at the base of the 5^th ^metatarsal. Treatment of fractures includes casting and orthopedic follow-up. Treatment of sprains follows the general principles of PRICE (protect, rest, ice, compression, elevation) and return to play guidelines (Table 2). Functional bracing (lace up brace, ASO), with early mobilization, is more effective than immobilization [12,13]. To protect the ankle from repeat or further injury, a brace is recommended for 3 to 6 months following return to activity and indefinitely in the case of persistent ankle instability. Chronic ankle instability requires continued physiotherapy exercises with a focus on balance and proprioception.

**Table 2:** Return to play guidelines.

1. Control pain and inflammation	PRICE (protect, rest, ice, compression, elevation)
2. Optimize range of motion	Active and passive exercises
3. Optimize strength	Isometric (static muscle contraction without joint movement), isotonic (constant muscle contraction with joint movement), dynamic and core strengthening exercises.
4. Optimize proprioception	Balance
5. Functional and sport specific skills	Running, jumping, pivoting etc.
6. Return to usual activities or sport	Practice before competitive play

#### Medial and syndesmosis sprains

An injury to the medial deltoid ligament is not as common as lateral ligament injuries and usually results from eversion stress to the ankle [14]. The deltoid ligament is stronger than the lateral ligaments and injury to the deltoid ligament may be accompanied by lateral complex injuries or fracture. A "high" ankle sprain refers to injury of the anterior inferior tibiofibular ligament and syndesmosis and can occur if the ankle is forced into dorsiflexion and external rotation. The external rotation test causes pain. Radiographs should be obtained for suspected medial or high ankle sprain and referral to sports medicine specialist considered. Treatment follows the same general principles for lateral ankle sprains but it may take 6 weeks or longer to rehabilitate. Disruption of the ankle mortise and some fractures require orthopedic open reduction and internal fixation.

#### Turf toe

Turf toe refers to sprain of the plantar capsule ligament. It is common in soccer and football players and usually occurs secondary to hyperextension injury of the 1^st ^MTP, with hyperflexion injuries less common. Painful restricted range of motion and localized swelling are common. Treatment includes RICE, non-steroidal anti-inflammatories (NSAIDS), early joint mobilization, toe taping, and increased rigidity of the shoe with a stiff insole. Long-term disability is not uncommon with decreased MTP range of motion, and degenerative arthritis (hallux rigidus) reported [15,16].

#### Tarsometatarsal (Lisfranc) injury

Tarsometatarsal fractures or fracture/dislocations are rare but may have serious complications if missed. Injury usually follows inversion stress to the ankle with force transmitted to the midfoot. Children with midtarsal joint sprains present with midfoot pain. Children with fractures or fracture/dislocations present with tenderness and swelling over the dorsum of the foot at the base of the second metatarsal and inability to weight bear. Suspicion of Lisfranc injury should prompt orthopedic referral. Treatment includes a period of non-weight bearing immobilization and possible surgical intervention [15].

### Overuse injuries and pain

#### Tendinopathies

Tendinopathies in the tendons crossing the ankle may be secondary to repetitive microtrauma from overuse, direct trauma and autoimmune disease including juvenile idiopathic arthritis (JIA). Muscle tendon imbalance may be altered by anatomic alignment, rapid growth, or change in activity level. Tendinopathies are painful with palpation, active use and resisted movement of the muscle-tendon complex.

Extensor tendinopathy occurs most commonly as an overuse injury in running athletes but may also be caused by compression from excessively tight shoelaces. Children present with pain over the tibialis anterior tendon as it crosses the ankle or aching in the dorsal midfoot with dorsiflexion.

Peroneal tendinopathy is an overuse injury commonly associated with excessive supination during toe-off while walking or running. It can also occur after ankle sprains. Children present with posterolateral ankle tenderness and swelling, painful snapping across the lateral malleoli pain on passive stretch with plantarflexion and inversion, and pain with resisted dorsiflexion and eversion. Subluxation of the peroneal tendons is not uncommon. Subluxation may be reproduced by the patient voluntarily circumducting the ankle or by palpating the lateral mallelous while the patient repeatedly dorsiflexes and plantarflexes the ankle.

Posterior tibialis tendinopathy is not common in skeletally immature athletes with the exception of association with symptomatic accessory navicular. Inflammatory tenosynovitis can occur in children with JIA. Children usually present with medial foot pain, tenderness and/or swelling along the tendon, pain on passive stretch with dorsiflexion and eversion, and pain with resisted plantar flexion and inversion.

Adolescents with Achilles tendinopathy usually present with pain with running and jumping activities and morning pain and stiffness. In Achilles tendinopathy pain is localized to the tendon and there may be pain with toe-raises and resisted ankle plantarflexion. Retrocalcaneal bursitis localizes tenderness to palpation anterior to the Achilles, proximal to the insertion on the os calcis. In addition to localized pain and swelling there may be stiffness with dorsiflexion and contracture of the Achilles tendon.

Management of tendinopathies includes RICE, activity modification, physiotherapy for flexibility and strengthening exercises, and orthotics. Management of Achilles tendinopathy includes modification of footwear with a gel heel cup or lift.

#### Stress fractures

Stress fracture occurs when normal stress is applied to abnormal bone or "abnormal" stress is applied to normal bone and represents a disturbance between bone resorption and bone regeneration. In the case of normal bones, it is felt to be a fatigue fracture or overuse injury.

Stress fractures are increasingly reported in the pediatric population. They are more common in youth who report a recent increase in activity, poor nutrition, and females with amenorrhea [17]. The second and third metatarsals are most commonly injured [18]. Injury to the proximal second metatarsal may include injury to the tarsal metatarsal (Lisfranc) joint and stress fracture may result in nonunion. Proximal fifth metatarsal stress fractures also have a high rate of poor healing. Navicular stress fracture is uncommon but important to recognize due to tenuous blood supply and poor healing. Children and adolescents usually present with progressively worsening pain, aggravated by weight bearing activity and relieved with rest. There may be associated swelling. On examination, the site of fracture is tender to palpation. Radiographs will detect chronic stress reaction; early lesions are best detected by MRI and radionucleotide bone scan. CT is useful for evaluation of high risk stress fractures (navicular, proximal second metatarsal, fifth metatarsal, medial mallelous). Management depends on symptoms and the site of injury. Treatment includes activity modification and if required a short period of non weight bearing and immobilization. Stress fractures of the navicular, proximal second metatarsal and proximal fifth metatarsal require sports medicine or orthopedic review. These high risk stress fractures often require non weight bearing immobilization for 6 to 8 weeks, and even then may not heal adequately [14,19]. Recent literature suggests faster and more regular healing, faster return to activity and lower risk of re-injury with early open reduction and internal fixation of proximal fifth metatarsal injuries [20,21].

#### Osteochondritis dissecans (OCD)

OCD is a lesion of bone and cartilage resulting in avascular bone necrosis and loss of continuity with subchondral bone. There may be partial or complete separation of articular cartilage with or without involvement of subchondral bone. Osteochondral lesions of the talus may cause chronic ankle pain. Adolescents often present with activity related pain, swelling and weakness. They may complain of catching, clicking or locking if a loose body is present. There is often history of remote macrotrauma (inversion ankle sprain) or repetitive microtrauma [22]. On examination there may be an ankle effusion, limited range of motion, pain over the anterolateral or posteromedial talus and not over the lateral ligament complex. Radiographs may show a radiolucent lesion, subchondral fracture, potential separation with subchondral bone and a loose body. Talar OCD lesions are best seen on mortise view but can be missed on routine radiographs. MRI may show cartilage changes earlier [7]. OCD lesions can be characterized by the radiographic appearance. The Berndt and Harty classification describes stage 1 lesions as a small area of compression, stage 2 as a separate fragment, stage III as a detached but hinged fragment and stage IV as detached fragments [23,24]. Treatment depends on the stage of the lesion, with earlier stage lesions having the best prognosis. Treatment options include modified activity with or without weight bearing; immobilization; cryotherapy; anti-inflammatories; drilling of subchondral bone to improve vascularity; and reattachment and/or removal of loose bodies [22].

#### Apophysitis

Apophysitis refers to inflammation at the tendon attachment. It is common during rapid growth due to physeal stress and may also be present with JIA. Sever's disease (calcaneal apophysitis) is a traction apophysitis of the os calcis at the insertion of the Achilles tendon. It usually affects children aged 7 to 14 [25]. Children usually present with pain that increases with running and jumping activities. On examination, there may be swelling and tenderness over the posterior heel at the insertion of the Achilles tendon, pain with medial and lateral squeeze test of the calcaneal apophysis, weakness of dorsiflexion and contracture of the Achilles tendon. Radiographs appear normal with normally irregular apophysis. Management includes RICE, NSAIDS, activity modification, Achilles stretching, strengthening of the ankle dorsiflexors, physiotherapy and insertion of heel lift or heel cup in footwear.

Iselin's disease refers to traction apophysitis of the tuberosity of the fifth metatarsal. The apophysis is within the peroneus brevis tendon insertion site. The secondary centre of ossification usually fuses by age 11 years in girls and 14 in boys. Pain with activity, tenderness over the base of the fifth metatarsal, and pain with resisted eversion are common. Radiographs differentiate Iselin's disease from avulsion fracture as the apophysis is parallel to the long axis of the fifth metatarsal and fractures are usually transverse [26]. Fractures at the metaphyseal-diaphyseal junction (Jones fracture) are significant injuries which require non-weight bearing immobilization and orthopedic referral. Technetium bone scan may help confirm apophysitis. Management includes RICE, modified activity, immobilization and physiotherapy.

#### Sesamoid pathology

The medial and lateral sesamoid bones act as pulleys for the flexor hallucis brevis tendons and help stabilize the first MTP joint. Sesamoid disease includes inflammation, fracture or sprain of bipartite sesamoid. Injury is usually seen in young athletes who repetitively push off the ball of their feet during activities such as jumping sports or ballet. Bipartite or multipartite sesamoids are present in 10 to 33% of feet [27]. Children and adolescents usually complain of pain with forefoot weight bearing and may walk with weight on the lateral border of the foot to compensate. On exam there may be localized tenderness and swelling Treatment includes RICE, NSAIDS, modified activity, modified footwear (wide toe box, stiff sole, low heel), orthoses (cut-out), and physiotherapy.

#### Plantar fascitiis

Plantar fascitiis is not as common in children and adolescents as adults. The plantar fascia aponeurosis stretches from the calcaneal tuberosity and divides to attach near the plantar aspect of the proximal phalanges and the base of the fifth metatarsal. It provides dynamic arch support. Plantar fascitiis is caused by repetitive microtrauma. In skeletally immature patients it may be associated with calcaneal apophysitis (Sever's disease). Enthesitis and periosteal inflammation associated with JIA (enthesitis related arthritis (ERA) subtype) and seronegative spodyloarthropathies can mimic plantar fascitiis. (See section on inflammatory disorders) In children with inflammatory disease, enthesitis is usually associated with tarsitis or peripheral joint arthritis and symptoms are more prominent in the morning. Children with plantar fascitiis usually report plantar heel pain worse with activity in addition to pain in the morning. On examination, tenderness is usually localized to the medial calcaneal tubercle with the foot in dorsiflexion. Tarsal arthropathy is characteristic of ERA. Investigations are helpful if systemic rheumatic disease is suspected. Diagnostic imaging is not useful for plantar fascitiis but may show erosive enthesitis in ERA. Treatment includes RICE, NSAIDS, modified activity, medial arch supports, physiotherapy, massage, fascial and Achilles stretching and strengthening.

#### Pain amplification syndromes

Many pediatric patients with severe chronic musculoskeletal pain do not have an identified cause. The cause of amplified musculoskeletal pain is unknown, but minor trauma, underlying chronic illnesses and psychological distress have been associated. This condition has been called many terms including reflex sympathetic dystrophy (RSD), reflex neurovascular dystrophy (RND), complex regional pain syndrome, and chronic musculoskeletal pain syndrome with or without autonomic dysfunction. Typically, an affected child sustains minor trauma to the lower extremity which results in pain out of proportion to the injury.

On physical examination, patients may be unwilling to weight bear, manifest allodynia (pain generated by usually non-painful stimuli), and autonomic changes such as excessive perspiration, edema, cyanosis, mottling, and coolness of the skin. General and neurovascular exam are normal. Diagnostic studies are typically not helpful. X-rays may show osteopenia but only in chronic cases with significant disability. MRI can show regional bone marrow edema, and a technetium bone scan may show decreased uptake. Treatment includes aggressive physiotherapy for desensitization, range of motion and function, and multidisciplinary team approach to help with pain coping mechanisms [28]. Immobilization makes the condition worse.

Medications (antidepressants, calcium channel blockers) or sympathetic nerve block may be helpful in recalcitrant cases. Typically, an affected child recovers from the initial episode but may have recurrence.

### Developmental and congenital conditions

#### Pes planus

Pes planus is a common presenting complaint to the primary care physician. Minimal or no medial longitudinal arch is common in children up to age 7 and occurs in 10–23% of the general population [29-31]. Presence of an arch while toe-standing or sitting and absent with standing suggests flexible flat feet. The majority of children with flexible flat feet are asymptomatic; parental concern is often the presenting complaint. Children with generalized hypermobility and ligamentous laxity who report pain after activity may benefit from a rigid medial arch support. In young athletes, excessive pronation while running may increase risk of ankle or knee injury and correction with orthotics may decrease this risk. Flat feet that are painful should be imaged to rule out anatomical variants including tarsal coalition, vertical talus or accessory navicular. Achilles contracture may result in progressive flat foot deformity or pain. Rigid flat feet have absence of the arch during toe-standing and sitting, and limited motion at the subtalar joint. Causes of rigid flat feet include anatomic variants such as tarsal coalition and arthritis of the subtalar joint.

#### Tarsal coalition

**Figure 3:**
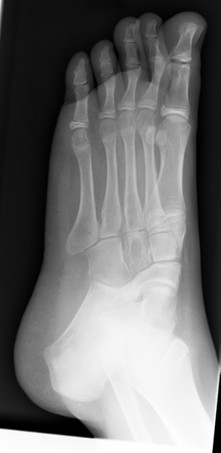
Standing lateral images of the left foot together with oblique images in this 10 year old with left flat foot clinically. Alignment of mid and hind feet looks to be normal. There is however evidence of calcaneal-navicular coalition on the left associated with both talar and calcaneal beaking. (Image courtesy of Robyn Cairns, MD).

**Figure 4:**
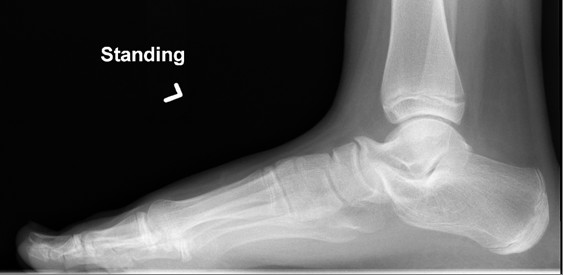
Standing lateral images of the left foot together with oblique images in this 10 year old with left flat foot clinically. Alignment of mid and hind feet looks to be normal. There is however evidence of calcaneal-navicular coalition on the left associated with both talar and calcaneal beaking. (Image courtesy of Robyn Cairns, MD).

Tarsal coalition represents failure of complete segmentation between two or more tarsal bones. It occurs in 1% of the population, affects boys twice as often as girls and is often bilateral. Calcaneonavicular and talocalcaneal coalitions are most common. Pain and symptom onset usually occurs at the time of ossification; 8 to 12 years for calcaneonavicular and 12 to 16 years for talocalcaneal coalitions [16]. Children may report a history of frequent "ankle sprains". Clinically, there is a rigid flatfoot with restricted and possibly painful subtalar range of motion Radiographs (oblique and axial views) may show calcaneonavicular coalitions (Figures 3 and 4) but CT or MRI is needed for fibrous or cartilaginous coalition and other tarsal coalitions [32]. Children with symptomatic coalitions require orthopedic consultation for casting and/or orthoses and physiotherapy or possible surgical excision of the bony bar.

#### Osteochondroses

Osteochondroses are unique to the growing skeleton and are felt to be due to repetitive stress and disruption of the vascular supply to bone. Freiberg's disease is an osteonecrosis of the metatarsal head. It commonly affects the second and third metatarsals and is more common in athletic adolescent females [27]. Adolescents usually complain of gradual onset forefoot pain that worsens with weight bearing activity. On examination there is focal pain and tenderness over the affected metatarsal head. Radiographs show initial widening of the MTP joint space, followed by collapse and sclerosis of the articular surface of the metatarsal head, and eventual reossification of the metatarsal head. The process takes 2 to 3 years. Management includes RICE, activity modification, modification of footwear and immobilization. Surgical management is reserved for those failing conservative strategies.

Kohler's disease is an osteochondroses of the tarsal navicular. It affects children between 5 and 9 years and may be bilateral in up to 25% of cases [27]. Children usually complain of midfoot pain that worsens with weight bearing activity. On examination there is localized swelling, erythema, and tenderness over the navicular. Radiographs show increased sclerosis of the navicular. Irregular ossification is also common in normal growth so clinical correlation is required for diagnosis. Management includes RICE, NSAIDs, activity modification, and immobilization in a walking boot for severe cases.

#### Accessory bones of the foot

Accessory navicular is the most common accessory bone in the foot, occurring in 4 to 14% of the general population [16]. A prominence may be visible in the proximal medial arch. It is usually asymptomatic but may become symptomatic with ossification. Adolescents may present with medial foot pain, synchondrosis disruption or posterior tibialis tendinopathy and dysfunction. On examination, adolescents complain of tenderness over the navicular. Resisted strength testing of posterior tibialis (plantar flexion and inversion) reproduces pain. Anteroposterior or 45 degrees eversion oblique radiographs confirm the presence of an accessory navicular. Technetium bone scanning may show localized increased uptake. Conservative treatment with physiotherapy and/or boot and rigid orthotics is generally successful. Surgical excision may be considered for recalcitrant cases.

The secondary ossification centre at the posterior aspect of the talus appears at age 8 to 10 years in girls and 11 to 13 years in boys. Failure to fuse creates an os trigonum. It is often unilateral and occurs in 10% of the population [16]. It can be congenital (persistent separation of secondary ossification centre) or acquired (fracture non-union). Most are asymptomatic; but children participating in sport involving repetitive ankle plantarflexion or inversion (soccer, ballet/en point, gymnastics) may complain of pain in the posterolateral ankle. On examination, there may be tenderness over the posterior ankle and calcaneus, and pain with forced plantarflexion (impingement of the posterior talus between the posterior tibia and calcaneus). Radiographs of the ankle, including lateral view in plantar flexion show the os trigonum. Pain due to posterior impingement may also be caused by soft tissues including synovitis, capsular hypertrophy and flexor hallucis longus tendinopathy. Management of os trigonum includes rest, NSAIDS, physiotherapy, taping and orthopedic referral for possible resection.

### Inflammatory disorders

Oligoarticular JIA often presents with ankle arthritis. Children may present with ankle or foot pain but the typical history of morning stiffness, gradual resolution of pain with activity and clinical exam findings of warmth, swelling and/or painful restricted range of motion usually allow the practitioner to make the correct diagnosis. A complete joint and systemic examination to exclude other joint involvement is important as is screening for asymptomatic uveitis associated with JIA.

The JIA subtype, ERA is unique in that it is more common in school-age boys, is frequently associated with the genetic marker HLA-B27 and inflammation of the entheses may be a prominent finding [33]. Enthesitis (inflammation at the attachment of tendons, ligaments and fascia to bone with characteristic pain on palpation) around the foot may be confused with Sever's disease or plantar fascitiis. Enthesitis is usually associated with arthritis and symptoms are more prominent in the morning, which helps to differentiate it from the osteochondroses and traction apophysitis group. Arthritis involving the subtalar and midfoot is more common in ERA and juvenile ankylosing spondylitis (JAS) than other childhood arthritides. Reactive arthritis and inflammatory bowel disease may also present with foot or ankle arthritis.

Treatment of arthritis includes physiotherapy for improving range of motion and strength, NSAIDS and potentially local intra-articular corticosteroid injection or disease modifying anti-rheumatic therapy. Custom made semi rigid foot orthotics with shock-absorbing posts may improve pain, gait, and function [34]. All children and adolescents suspected of having JIA or other chronic inflammatory arthritis should be referred to a pediatric rheumatologist.

### Tumors

Benign and malignant tumors are rare causes of foot and ankle pain. Symptomatic benign bone lesions include osteoid osteoma and non bacterial chronic osteomyelitis (previously called chronic recurrent multifocal osteomyelitis). Malignant bone tumors include local osteosarcoma or Ewing's sarcoma, leukemia or metastases from neuroblastoma. Benign synovial tumors include pigmented villonodular synovitis (PVNS) and hemangioma. Malignant synovial tumors include sarcomas.

## Conclusion

Foot and ankle pain are common in the pediatric population. Pain can usually be localized to the forefoot, midfoot, hindfoot or ankle. Painful conditions are often related to skeletal maturity and are fairly specific to the age of the child. It is important for the clinician to be aware of conditions unique to the growing pediatric skeleton including congenital and developmental variation (pes planus, tarsal coalition, os trigonum, symptomatic accessory navicular, hallux valgus), apophysitis and osteochondroses (Sever's disease, Iselin's disease, Kohler's disease, Freiberg's disease), skeletal maturation (growth centers, growth plate fractures) and lower extremity alignment. Foot and ankle pain is occasionally caused by serious underlying systemic disease including inflammatory conditions and malignancies. The clinical history and physical exam often lead to correct diagnosis and identify cases requiring further evaluation, imaging or referral. Identification and treatment of predisposing biomechanical factors is important to reduce symptom recurrence. General return to play guidelines can be followed for most causes of foot and ankle pain. Children and adolescents with an unstable ankle require referral to a sports medicine specialist.

## Competing interests

The author(s) declare that they have no competing interests.
